# Development of the breast immobilization system in prone setup: The effect of bra in prone position to improve the breast setup error

**DOI:** 10.1002/acm2.12116

**Published:** 2017-06-08

**Authors:** Mariko Kawamura, Yoshikazu Maeda, Kazutaka Yamamoto, Shigeyuki Takamatsu, Yoshitaka Sato, Hiroki Minami, Yusuke Saga, Kyo Kume, Yuji Tameshige, Makoto Sasaki, Hiroyasu Tamamura, Kouji Ohta, Yoshiyuki Itoh, Shinji Naganawa

**Affiliations:** ^1^ Department of Radiology Nagoya University Graduate School of Medicine Nagoya Aichi Japan; ^2^ Proton Therapy Center Fukui Prefectural Hospital Fukui Japan; ^3^ Department of Radiotherapy Graduate School of Medical Science Kanazawa University Ishikawa Japan; ^4^ Research and Development Division The Wakasa Wan Energy Research Center Fukui Japan; ^5^ Department of Surgery Fukui Prefectural Hospital Fukui Japan

**Keywords:** breast, immobilizing bra, prone, setup error

## Abstract

**Purpose/objective(s):**

Accurate and reproducible positioning of the breast is difficult due to its deformability and softness; thus, targeting a breast tumor or tumor bed with fractionated radiotherapy using external beam radiation is difficult. The aim of this study was to develop a novel bra to aid in breast immobilization in the prone position.

**Materials & methods:**

To assess the accuracy of prone position fixation of breast tumors, 33 breast cancer patients with 34 lesions were recruited. The bra used in this verification was customized from a commercially available bra. Duplicate MRI were acquired in the prone position, alternating with and without the bra, and for each series, patients were asked to step off the MRI table and re‐set up in the prone position. Patients were also asked to remove and re‐fit the bra for the second MRI. Each pair of images were superimposed to match the shape of the skin surface, and the maximum difference in tumor geometric center in three axes was measured. The required set up margin was calculated as: required margin = mean difference in geometric center + 2.5 standard deviation. The volumetric overlap of the tumor, as well as contouring uncertainties, was evaluated using contour analysis software.

**Results:**

The median breast size was 498 cc. The required margins for the lateral, vertical, and longitudinal directions were estimated to be 4.1, 4.1, and 5.0 mm, respectively, with the bra, and 5.1, 6.9, and 6.7 mm, respectively, without the bra. These margins covered the dislocation of more than 33 lesions in total. With the bra, 33 lesions had achieved an objective overlap of 95% and 99% with 2 and 4 mm margins, respectively, whereas 4 and 8 mm, respectively, were needed without the bra.

**Conclusion:**

The use of an immobilizing bra reduced the setup margin for prone position fixation of breast tumors.

## 1 INTRODUCTION

The efficacy of breast‐conserving therapy (BCT) in the treatment of early‐stage breast cancer has been established through multiple randomized trials.^1^, ^2^ Furthermore, because most local recurrences appear close to the lumpectomy cavity, accelerated partial breast irradiation (APBI) in highly selected patients with low local recurrence‐risk, achieved local control comparable with that of whole breast irradiation (WBI).^3^, ^4^ APBI has gained popularity in recent years because it may shorten the treatment course. However, because the irradiation field is smaller and a fractional dose is higher with reduced number of fractions, in which result in fewer opportunities to compensate for geometric errors for APBI, an accurate and reproducible positioning in everyday treatment is essential.

Some reports highlight the utility of prone positioning for WBI, with the aim of minimizing breast deformation, especially for patients with large breasts.^5^ The prone technique also decreases the breathing motion of the chest wall, thus minimizing intra‐fraction motion.^6^, ^7^ Currently, image‐guided radiotherapy technology has made a great improvements, permitting visualization of computed tomography (CT) or even magnetic resonance imaging (MRI) during treatment. However, visualizing the tumor itself, or the tumor bed of the breast cancer without the use of contrast‐enhancing media remains challenging, and deformation of the tumor bed is a critical factor when accurately determining the PTV margin to the tumor bed.

The aim of this study was to develop a novel bra to aid in breast immobilization in the prone position, and to analyze the accuracy of tumor immobilization when matched by the shape of the skin surface.

## 2 MATERIALS AND METHODS

An institutional review board approved this study, and all patients gave written informed consent. Patients with invasive breast cancer considering conservative surgery, who underwent MRI prior to the surgery between December 2011 and November 2013 were recruited to this study. The study was performed just after pre‐operative magnetic resonance imaging (MRI) was obtained.

### 2.A MRI procedure

The study was performed just after undergoing a pre‐operative enhanced MRI. The magnetic resonance apparatus used was a 1.5 T system (Signa HD × 1.5 T Optima Edition, General Electric Healthcare, Waukesha, USA). Patients were scanned in the prone position with a dedicated four‐channel, phased‐array, bilateral breast coil. The parameters of the sequence were: TR/TE 5.3/2.5 ms, flip angle 12 deg. FOV 300 × 300 mm, matrix 320 × 320, thickness 1.6 mm, acquisitions 3D VIBRANT, acquisition time 128 s. Duplicate MRI images were acquired in the prone position, alternating with and without the bra (orders of images were: (a) without bra, (b) with bra, (c) without bra, and (d) with bra), and for each series, patients were asked to step off from the MRI table and re‐set up in the prone position.

### 2.B Bra setting

**Figure 1:**
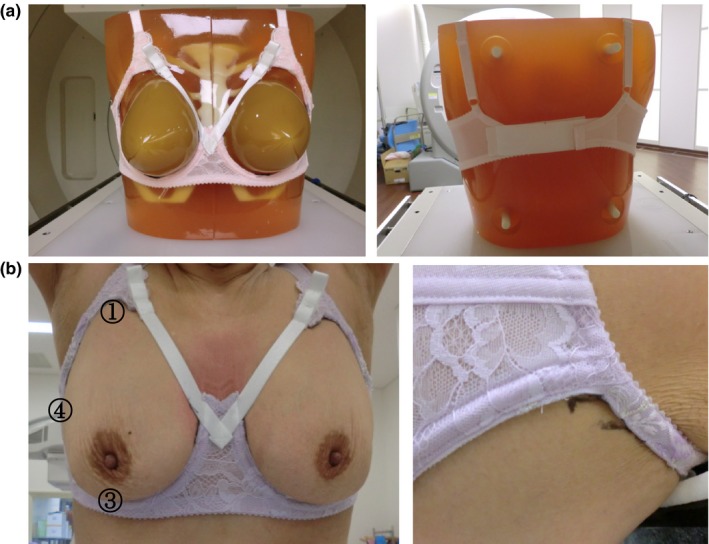
(a) Remaking the bra: The covering cloth of bra cup was removed and the underwire of the cup was replaced with plastic material. The cup joint and the straps were fastened with strap material (left), and the metal hooks were removed from bra and replaced with Velcro tape (right). (b) Procedure of wearing the bra and the skin marks. The patient wears the bra as usual. The strap is then adjusted with the arms in a raised position. Marks 1–4 were drawn by felt tip pen (left) on the skin to guide the next set up. Mark 4 usually drops in the prone position, so a new mark was drawn on the skin in the prone position (right).

The bra used in this study was modified from a commercially available bra (BRB228 13X05CC, BRG228 13X01GW; Wacoal co., Japan) that is used as non‐medical device. For the redesign of the bra, the covering cloth of the bra cup was removed, and the metal underwire of the cup was replaced with plastic material to reduce the artifact from metal while acquiring images. The cup joint and the straps were fastened with nylon strap material, and the metal hooks were removed from the bra and replaced with Velcro tape [Fig. 1(a)]. The patients wore the bra as they usually would, and the straps were adjusted with the arms in the up position so the cup would not move with arm motion. The bra setting was marked using a felt tip pen at the fixed position of Velcro tape and on the skin at four points: superior, inferior, medial, and lateral parts of the cups [Fig. 1(b)], and on the right and left breast just prior to the first MRI acquisition with the bra on. After wearing the bra, patients were set on the MRI table in the prone position. Because gravity causes the skin mark on superior and lateral breasts to drop in the prone position, an additional skin mark was drawn in the prone position.

The inferior mark usually did not move with the patient's position. However, if it did, then re‐adjustment of the strap and Velcro tape was required. Patients wore the bra according to the standard skin markings for the second fitting.

### 2.C Tumor immobilization analysis

Contour analysis software (MIM maestro v6.5, MIM software Inc., USA) was used for the tumor immobilization analysis. The breast size was defined as the entire ipsilateral breast tissue starting 5 mm below the skin. The tumor was delineated in each pair of MRI images with and without the bra by one radiation oncologist, with more than 5 yr experience in the field, as regions of interest (ROI). To assess the uncertainties of the tumor delineation, ROI size by maximum distance in the x, y, and z axes and ROI volume were compared in each patient. In addition, each tumor in MRI images, with and without the bra was recreated 2 yr later by the same oncologist. The variations in the volume and the displacement of the geometric center along three axes coordinates were also evaluated among the 34 lesions.

Pairs of images with and without the bra were superimposed respectively to match the shape of skinsurface by two therapists, with more than 3 yr experience each, without seeing the ROI. This procedure was repeated 2 yr later thrice by the same therapists to assess the setup uncertainties by therapists. Furthermore, we also used the edge detection fusion mode of the MIM v6.5 software for automated fusion of multiple images. In the software fusion mode, a box was created to cover the desired target, in this case the ipsilateral breast, resulting in the automatic fusion of the two images.

The change in the ROIs for each lesion were defined as the differences in the geometric center of each ROI, and were evaluated with respect to three axes (anterior‐posterior [AP]; superior‐inferior [SI]; medial‐lateral [ML]). The mean value and the standard deviation (SD) for the differences were estimated with and without the bra. The required setup margin was calculated as: Required margin = mean difference of the geometric center + 2.5 SD.

Volumetric overlap of the ROI was also evaluated using the equations of $\left(V_{1m}\cap V_{2}\right)/V_{2}$, where $V_{1m}$, and $V_{2}$ show the ROI with the margin of the early, and delayed images, respectively. Margins from 0 to 10 mm (1 mm per step) were set to the early ROI. The number of lesions with an overlap ratio ˃95% was checked to validate the three dimensional margin evaluations for all lesions with and without the bra.

## 3 RESULTS

Thirty‐three women with 34 lesions among them participated in the study. Their breast volume ranged 156–1,432 cc (median 498 cc), and the tumor volume ranged 0.5–47.2 cc (median 5.8 cc).

**Figure 2:**
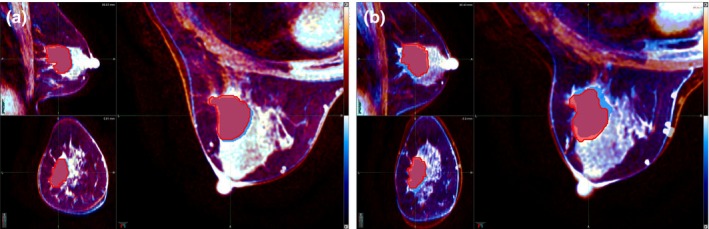
The early and delayed MRI‐images superimposed (a) with the bra and (b) without the bra, for patient ID18. The orange and blue contrast images correspond to the early and delayed images, respectively.

Figure 2 shows the early and delayed images superimposed for patient identification number 18 (a) with and, (b) without the bra, respectively.

The volumes of the early and delayed ROI, and re‐evaluation performed 2 yr later were concordant, and were within 10% of one another in all 34 lesions. However, in the case of the volumetric difference without the bra, the mean values deviated from the baseline by about −4%, which means that the early volume was likely to be smaller than the delayed volume. In the early phase, tumor enhancement represents enhancement of the vasculature, while stromal enhancement in the tumor is captured at the delay phase. Therefore, delay volumes are likely to be larger than the early phase. When the ROI shape was converted to a sphere with equivalent volume, the difference of the diameter was within 0.5 mm. However, the size differences by means of three axis maximum extent of ROI were found to be 1.2–1.9 mm on average, which was larger than when the bra was worn. These were accommodated by implementing both a delineation procedure, and target deformation, and were considered to be the uncertainties for margin estimations.

**Figure 3:**
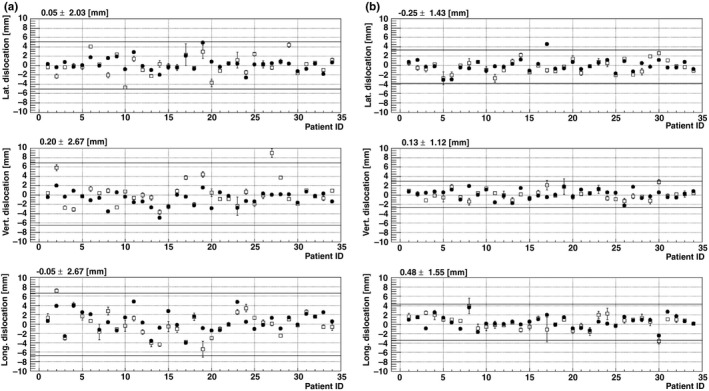
The change in the ROIs in three axes are shown, (a) without (b) with bra, for all 34 lesions. The average deviation by manual registration is represented by □, and the deviation due to auto registration is represented by ●. The line in each graph represents mean + 2.5 SD

The deviations of the ROI by manual registration and auto registration in all cases were as shown in Fig. 3, where horizontal lines represent the value of the mean ± 2.5 SD. The manual registration data represent the mean value from 4 exams with its range. Patient #30 had greater deviations with the bra than without. Her breast size was the smallest among all patients, and the bra did not fit well that her breast deformed more with the bra than without. The mean and SD of ROI dislocations in three axes were as shown in Table 1.

**Table 1:** The mean and standard deviation of region of interest deviations in three axes

	Without bra (RMS)		With bra (RMS)		R	
	Auto	Manual	Auto	Manual	Auto	Manual
Lateral	1.4	2.0	1.4	1.5	1	0.8
Vertical	1.5	2.7	1.0	1.6	0.7	0.6
Long	2.2	2.7	1.3	1.8	0.3	0.7

Although the deviations along each direction were scattered, depending on the value of the SD, the mean values were within 0.5 mm of one another. The values of the SD with the bra were lower than that of the value without bra, by a factor of 0.8, 0.6, and 0.7 for the lateral, vertical, and longitudinal directions, respectively, if registered manually. Required margins for lateral, vertical, and longitudinal directions were estimated to be 4.1, 4.1, and 5.0 mm, respectively with the bra, and 5.1, 6.9, and 6.7 mm without the bra, if registered manually. The required margin would change to 3.4, 2.8, and 3.7 mm with the bra, and 3.9, 4.5, and 6.0 mm without the bra if registered automatically. These margins could cover the deviation of more than 33 lesions in total. Table 2 shows the transition of the number of lesions achieving the objective volumetric overlap as the adapting margins to the early ROI are increased from 0 to 10 mm. With the bra, 33 lesions in total had achieved an objective overlap of 95% and 99% with 2 mm and 4 mm margin, whereas 4 mm and 8 mm needed to be adapted without the bra. The margin evaluated for the objective overlap of 99% was very nearly concurrent with the required margin estimated by the deviation analysis.

**Table 2:** The relations of volumetric overlap ratio of the region of interest with margins from 0 to 10 mm

mm	0	1	2	3	4	5	6	7	8	9	10
(a) Volume overlap ratio > 95%											
without bra	0	8	17	26	30	30	31	32	33	all	all
with bra	0	25	33	33	33	all	all	all	all	all	all
(b) Volume overlap ratio >99%											
without bra	0	2	9	19	25	29	30	30	33	33	33
with bra	0	4	24	32	33	33	all	all	all	all	all

## 4 DISCUSSION

The known advantage of prone positioning is to reduce skin folds and respiratory motion while moving the target tissue away from the chest wall, especially to the patients with large breasts.^5^, ^7^ The known disadvantage of prone positioning is its difficulty in visualizing the light fields, which plays an important role in whole breast irradiation. However, those advantages and disadvantages are essentially focused solely on whole breast irradiation. One has to consider the setup uncertainties of the target inside the breast, in this case the tumor, or tumor bed, and as such, we have to consider the motion and deformation of the target itself as well as the motion and deformation of the whole breast. As we all know, the breast itself is very soft and easily deforms, thus targeting the tumor or tumor bed with external irradiation is very difficult. Leaving surgical clips inside the lumpectomy cavity is one means of reproducing the target for external beam irradiation.^8^ However, if the cavity itself deforms, the patient cannot achieve the dose that was planned in the radiotherapy planning procedure. To solve this problem, we evaluated the tumor, and thus we were able to evaluate the target as a volume, permitting deformation of the target to be considered.

The uncertainty in tumor bed delineation has been criticized in many reports.^9^ We therefore used pre‐operative MRI, and as such, the uncertainties arising from delineation of the target were minimal, and there were no differences in either the size or volume of the tumor with or without the bra.

First, we analyzed the differences in the geometric center of the tumor with manual registration by two therapists, because this is the most common method in practice. However, this procedure is highly subjective, and was therefore re‐evaluated 2 yr later. Furthermore, the same procedure was performed in triplicate to see the range of error, and an auto‐recognition module in the commercially available contour analysis software was used to objectively analyze the two images using the MRI signal threshold. In the procedure of auto‐recognition, the ipsilateral breast was selected in the software, and the threshold was set to where the skin surface could clearly be captured. However, in the threshold set in the auto‐recognition module in the software, structures inside the breast, especially the contrasted tumor, remained visible, thereby affecting the result. With auto‐recognition module, the software could have registered two images considering enhanced tumor as well as skin, where with manual registration, we tried to register by just skin surface. And we believe this may be one of the reasons why the tumor dislocation was minimal with auto registration than manual procedure. Furthermore, from a therapist's perspective, since skin deformation was minimal with bra, the matching procedure of the shape of the skin surface was easier with the bra.

The highlight of our study is that we used a commercially available bra. For the daily treatment of common cancers, it is very important to provide a simple and inexpensive method. It is much less expensive to use a commercially available bra than to create completely new devices. Furthermore, commercially available bras come in many different types and sizes, thus it is very easy to find one that best suits the patient. However, we do recommend soliciting help from bra manufacturers in the customization of the bra. We used a bra with a metal underwire, thus, we had to replace the wire to plastic material so that we will not have any artifact in the planning CT or MRI. The main reason for using the bra is to keep the skin from dropping freely and to achieve a reproducible breast skin position. Thus, the procedure of superimposing the skin surface was much easier for the groups with the bra as well.

The major limitation of our study may be the fact that we evaluated intra‐fractional deformation in 1 day, so that breast's daily deformation could not be considered. To minimize the variability in contouring, we decided to use pre‐operative MRI and used the tumor as the target. The target delineation was performed based on contrasted enhanced MRI, but due to washout of the contrast media, taking more than three diagnostic MRI series remains a challenge. In addition, because the patients underwent MRI prior to the surgery, it was difficult to re‐administer MRI before the surgery, on another day especially with additional unnecessary contrast media. Although we failed to evaluate the reproducibility in multiple fractions, the main scope of this paper was to assess the deformation of the structures inside the breast relative to the skin surface. Furthermore, daily deformation of the breast is not vast in post‐menopausal women, and the majority of the patients considering APBI are elderly, or post‐menopausal women.^10^ Thus, the effects from differences in daily breast size would be limited. Another limitation may be the fact that the breast sizes of the tested patients were relatively small, because Asian women tend to have smaller breasts compared to Western women;^11^ thus, the average required margin may not be appropriate for a Western population. In addition, as seen in patient #30, women with breasts size <200 cc may not require bra at all.

Before applying this method of using a modified bra to improve set up error in clinical practice, one must consider how the bra effects the multi‐fraction irradiation by means of set up error and breast swelling, and further, how the contralateral breast may affect the set up. MR‐linac imaging, or in‐room CT may provide an answer to these questions in practice.

## 5 CONCLUSION

In summary, we analyzed how a modified bra can help to immobilize breasts in the prone position for MRI set up, affecting both the skin and the tumor. Further investigation may be necessary to calculate PTV margin if the mean breast size is larger than that of our study.

## ACKNOWLEDGMENT

This work was supported by Fukui prefectural government with the grant for nuclear power plant areas from Agency for Natural Resources and Energy, Japan. Also, special thanks to the Wacoal company for their great contribution in remaking the bra.

## CONFLICT OF INTEREST

None of the authors have any financial interest or personal relationships with other persons or organizations that could inappropriately influence our work.
